# A methodological review on material growth and synthesis of solar-driven water splitting photoelectrochemical cells

**DOI:** 10.1039/c9ra05341g

**Published:** 2019-09-23

**Authors:** Kwangwook Park, Yeong Jae Kim, Taeho Yoon, Selvaraj David, Young Min Song

**Affiliations:** Division of Advanced Materials Engineering, Jeonbuk National University Jeonju 54896 Republic of Korea; School of Electrical Engineering and Computer Science, Gwangju Institute of Science and Technology Gwangju 61005 Republic of Korea ymsong@gist.ac.kr; School of Chemical Engineering, Yeungnam University Gyeongsan Gyeongbuk 38541 Republic of Korea

## Abstract

As a renewable and sustainable energy source and an alternative to fossil fuels, solar-driven water splitting with photoelectrochemical (PEC) cell is a promising approach to obtain hydrogen fuel with its near-zero carbon emission pathway by transforming incident sunlight, the most abundant energy source. Because of its importance and future prospects, a number of architectures with their own features have been formed by various synthesis and growth methods. Because the materials themselves are one of the most dominant components, they determine the solar-to-hydrogen efficiency of the PEC cells. Thus, several representative PEC cells were reviewed by categorizing them as per synthesis and/or growth methods such as physical vapor deposition, chemical vapor deposition, electrochemical deposition, *etc.* This review provides researchers with an overview and acts as a guide for research on solar-driven water splitting PEC cells.

## Abstract 2

Solar-driven PEC cell is a promising approach to obtain hydrogen with near-zero carbon emission pathway. In this article, PEC cell was reviewed as per growth/synthesis methods. This review provides an overview and a guide for research on PEC cell.

## Graphical abstract

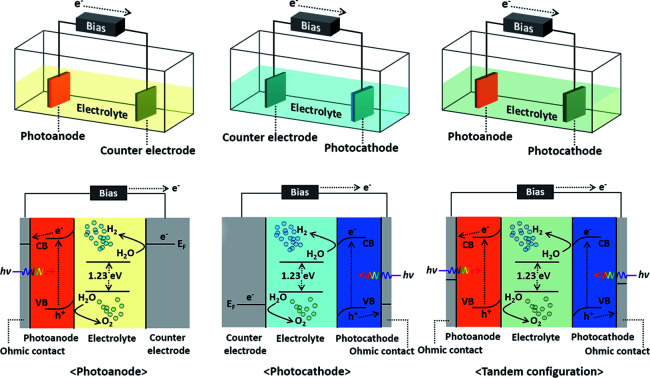

## 1. Introduction

With the growing importance of hydrogen as a near-zero carbon emission fuel,^1^ a number of hydrogen production methods from various approaches and sources have been emerging and highlighted for decades.^2^ The hydrogen production technologies reported to date can be categorized into the following four different processes depending on the driving force of the hydrogen production. These processes are thermochemical, electrolytic, biological, and photolytic.^3^ Cracking petroleum or reforming natural gas with steam methane are examples of thermochemical processes and are mainstream in hydrogen production comprising 95% of the global production.^4^ However, these methods are not favourable due to unnecessary emission of carbon dioxide during the hydrogen production.^5^ In the electrolytic process, water is split directly into hydrogen and oxygen by electricity in electrolytes. However, carbon dioxide emission during hydrogen production depends on the energy source of the electricity. If the electricity is generated by carbon-free energy sources such as wind, hydro, or solar, then we can consider the electrolytic process as a carbon-free pathway for hydrogen production. However, if the energy sources for electricity generation for hydrogen production are natural gas, propane, coal, or methane, then we cannot say the process is a carbon-free method at all.^6^ The biological process turns a biomass into hydrogen and by-products through a microbial process called anaerobic digestion.

It does less likely require fossil fuels among the four different processes; however, the efficiency is very low. Additionally, some microorganisms still produce carbon dioxide along with hydrogen and oxygen.^7^ Based on these perspectives of the above-mentioned methods, the photolytic process or solar-driven hydrogen production is the most suitable for the near-zero carbon industry and most environment friendly technology.^8^ This is because water splits into hydrogen and oxygen simply by incident solar energy, which is the most abundant source of energy, without any intermediary such as electricity in the photolytic process. Solar-driven hydrogen production is an active area with the aim toward a sustainable energy economy. Because of this issue, many countries have announced hydrogen fuel policies recently.^9^ The United States Department of Energy has a target cost of 2 USD per kg for hydrogen production from solar-driven fuel cells.^10^ The Netherlands also launched a 25 million-euro project, Toward BioSolar Cells, a consortium of six universities working on solar fuel production. UK and Switzerland also launched a project called Solar Hydrogen Integrated Nanoelectrolysis (SHINE). France also announced a target occupancy of overall hydrogen production from 10% in 2022 to 40% by 2027.^11^

**Fig. 1:**
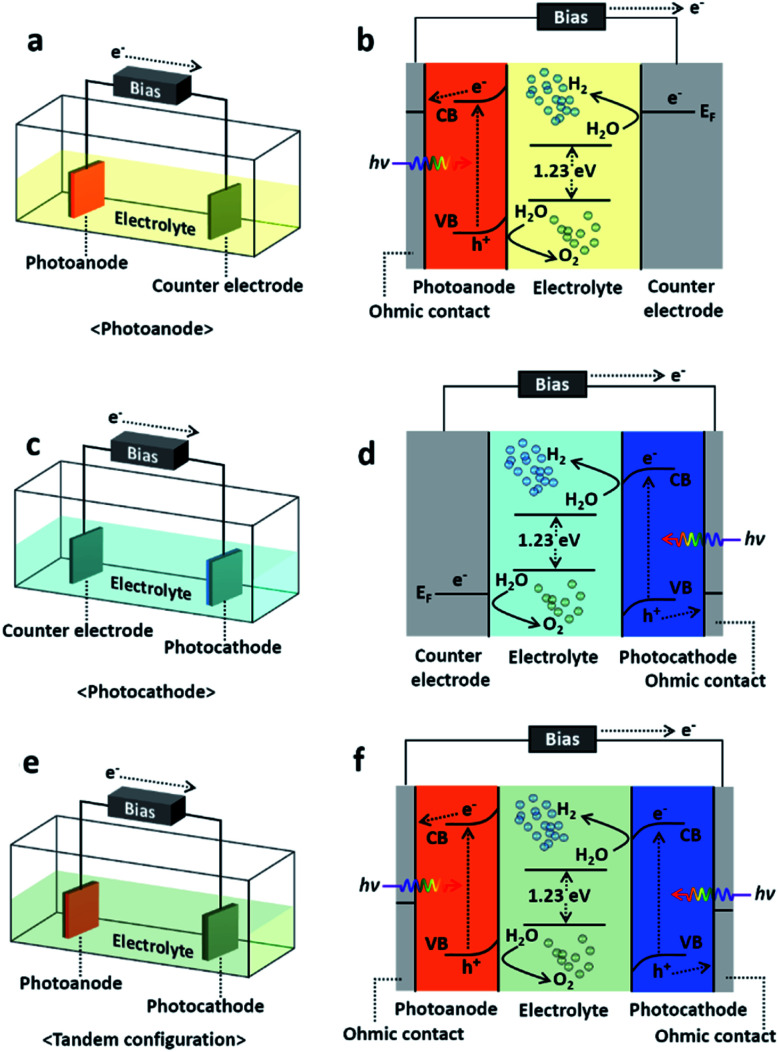
Schematics and energy diagram of PEC water splitting cells. (a), (c), and (e) shows schematic of schematics of PEC cell configurations of photoanode, photocathode, and tandem configuration (*Z*-scheme) PEC cells. (b), (d), and (f) displays their detailed mechanisms in energy band.

Since the first report by Akira Fujishima and Kenichi Honda,^12,13^ solar-driven hydrogen production has provided a way to increase the solar-to-hydrogen (STH) conversion efficiency and to reduce the fabrication cost.^14^ The photoelectrochemical (PEC) process, one of the methods of solar-driven hydrogen production, was first observed with a crystalline n-type TiO_2_ semiconductor. With the incident light, water is oxidized electrochemically with the electrons excited due to the band gap energy of the TiO_2_. A semiconductor material that can cause an electrochemical reaction by light illumination such as water splitting is called a photocatalyst, and the reaction itself with photocatalytic electrodes is called a photoelectrochemical reaction. PEC water splitting can only occur once the energy delivered to the PEC cell is higher than a Δ*G* of 237.2 kJ mol^−1^ (or a potential of 1.23 eV per electron), which are the changes in Gibbs free energy for converting one molecule of H_2_O into H_2_ and 1/2 O_2_ under standard conditions.^15,16^ In principle, the PEC water splitting process follows the following procedure: (1) light absorption, (2) electron–hole charge separation, (3) redox reactions, and finally (4) adsorption/desorption.^17^ However, along with the ideal whole water splitting, recombination of photogenerated electron–hole charges also occurs during the photoelectrochemical reaction and is the origin of the loss in conversion from solar energy to hydrogen fuel. Details of the PEC water splitting mechanism and fundamental issues can be found in ref. 17–19, 83 and 84. The PEC cell is basically composed of an electrolyte, semiconductor photoelectrode and its metallic counter electrode. The photoelectrode can be either a photocathode (p-type semiconductor) or photoanode (n-type semiconductor) which transfers electrons through the electrolyte solution so that the Fermi level of the photoelectrode reaches equilibrium with the redox potential of the electrolyte.^20^ If the photoelectrode is n-type semiconductor, *i.e.*, a photoanode, then the holes excited due to the incident light oxidize the water producing oxygen on the photoanode surface, and the electrons are transferred to the counter electrode generating hydrogen. In a similar regime, when it comes to a p-type photocathode, water is oxidized on the counter electrode surface generating oxygen, and the photocathode produces hydrogen.^16^ In addition to the photocathode and photoanode, the PEC cell typically falls in one of three categories shown in Fig. 1, which are photoanode, photocathode, and tandem configuration consisting of both a photoanode and photocathode, so called *Z*-scheme. In general, the photoanode or photocathode couples with the counter electrode and utilizes external bias for single-side water oxidation to prevent a potential deficiency and thus accelerating the charge separation. On the other hand, the tandem configuration is the combination of a wired photoanode and photocathode thereby promoting the photoexcitation of electrons and holes, respectively. It typically is self-biased with the help of the direct integration of p-type photocathode and n-type photoanode semiconductors with different Fermi levels.^21^ In this article, we will not focus on the PEC cell classification but the formation methods instead. Indeed, choosing a suitable material and/or the proper combination of multiple materials itself is a key factor in renewable and sustainable energy applications including the materials for PEC cell applications.^22^ A recent analysis proposed that a PEC cell should have a STH efficiency higher than 10% sustaining a long lifetime in pure water to meet economical requirements.^23^ Nevertheless, the PEC cells reported to date have either a high STH efficiency or a low fabrication cost but do not satisfying both conditions. For example, solution-based processes are environmentally friendly, easily scalable, and cost effective^85^ nevertheless the efficiency of the PEC cell formed through solution-based process is relatively low. From this perspective, again choosing the proper material for PEC cells is absolutely necessary, and thus, understanding the methods to synthesize and/or grow them is essential. A recent review reported on the requirements for photoelectrode materials.^24^ They were as follows: (1) a sustainable band gap energy and band positions, (2) an efficient change carrier separation and transportation, and (3) a strong catalytic activity and stability, surely fabricated with abundant elements for practical uses and low-cost. In these terms, we will take a look at PEC cells as per synthesis or growth method in four different categories which are physical vapor deposition (PVD), chemical vapor deposition (CVD), electrochemical deposition (ECD) and other techniques which cannot be categorized clearly, despite to define a PEC cell just simply with a single method is barely possible. Nevertheless, classifying, reviewing the synthesis/growth methods one by one by categorizing the PEC cells should provide guidance to researchers who have an interest in solar-driven water splitting with PEC cells.

## 2. Growth and synthesis methods

### 2.1 Physical vapor deposition

A key feature of the PVD process, which is distinguished from the others, is the absence of chemical reactions and organic ligands as surfactants during thin-film deposition.^25^ Moreover, several required conditions for the PVD process such as a high-vacuum chamber system, high growth/synthesis temperature, and use of evaporated solid sources by physical means such as plasma, electron beam, heat, laser and so on are other features of the PVD process.^14^ With these features, a PVD grown thin-film usually has a high purity and quality and is known to be corrosion resistive compared with the ones formed through the other techniques. These characteristics of PVD grown thin-films make this method favourable for the growth or synthesis of a variety of semiconductors. However, with the requirements of a high temperature and a high-vacuum atmosphere for this process, it has a relatively high cost and a complicated system configuration compared with the solution-based methods and the CVD methods. There are many PVD methods available including sputtering, evaporation process, pulsed laser deposition (PLD), molecular beam epitaxy (MBE), *etc.* The crystallinity of the PVD grown thin-films are typically polycrystalline, but single crystalline thin-films are also achievable with MBE.

**Fig. 2:**
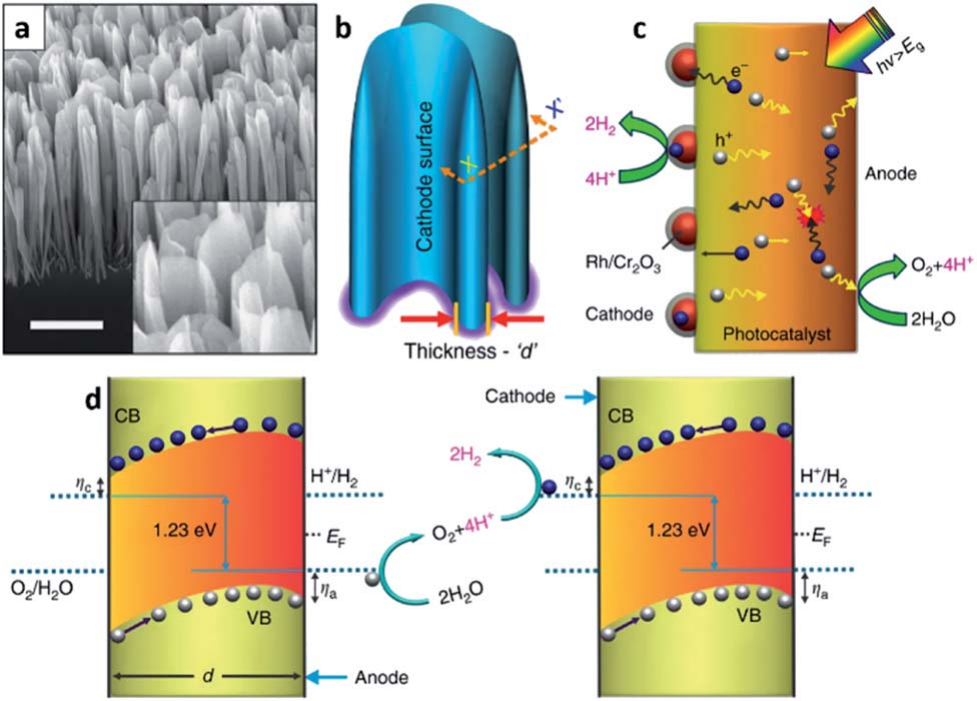
Reconstructed image from ref. 30. (a) A 45° tilted SEM image of InGaN nanosheets vertically aligned on a Si substrate. Scale bar, 1 μm. (b) Schematic illustration of probing the nanosheets with an arbitrary thickness *d*. (c) Depiction of the dynamic behaviors of the charge carriers in a single-photon InGaN nanosheet upon photoexcitation. The electron enriched surface (cathode) of the nanosheet is largely decorated with a photo-deposited hydrogen evolution reaction (HER) co-catalyst. (d) Neutral pH overall water splitting on the surfaces of InGaN nanosheets, presented schematically as a top view at the plane (*X*–*X*′) of the cross-section in (b). *η*_a_ and *η*_c_ represent the anodic and cathodic over-potentials for the water oxidation and proton reduction reaction, respectively. With the directional (opposite) migration of electrons and holes, redox reactions can be coupled between parallel (cathode and anode) surfaces of vertically aligned adjacent nanosheets. Reprinted with permission from ref. 30. Copyright 2018 Springer Nature.

MBE refers to a method growing single crystalline thin-film, *i.e.*, an epitaxial layer, in an ordered manner and on an atomic scale using high purity vaporized source materials from Knudsen cells in ultra-high vacuum (UHV) chambers.^26^ To maintain the UHV environment in the system, the main chamber is separate from the preparation and load-lock chambers which are equipped with UHV and/or HV pumps such as a cryogenic pump, ion pump, and turbo pump, *etc.* With this system configuration, it is possible to obtain excellent quality thin-films. Indeed, crystallographic properties have an important role in solar-driven water splitting PEC cells.^27^ Materials with poor quality and a high defect density typically have a number of defect states between the conduction and valence band edges, and they become radiative or non-radiative recombination centers. As a result, separation and transfer of photo-generated charges are inhibited thereby reducing the STH efficiency of the PEC cell. From this perspective, MBE is advantageous in growing PEC cells with a high STH efficiency and excellent crystalline quality.^28^ However, this condition is valid only if the thin-film grown and the substrate are closely lattice matched. Once the thin-film for the PEC cell is highly lattice mismatched with the substrate beneath, then the formation of high-density defects becomes inevitable. Fortunately, this issue can be avoided by growing nanostructures instead of planar thin-films. Unlike thin-films, the strain relaxation of nanostructures hinders defect formation because of their low dimensionality.^29^ A large surface-to-volume ratio is also another advantage of nanostructures when it comes to PEC cells due to the increased surface area which can enhance light absorption and surface reactions. Faqrul A. Chowdhury *et al.* recently reported on a PEC cell with vertically aligned InGaN nanosheets grown by MBE (Fig. 2).^30^ InGaN is a material whose band gap energy can be tuned in the range of nearly the entire solar spectrum and thus, can straddle the water redox potential in any spectral range.^31^ The band gap tunability along with other properties such as suitable band edges for overall water splitting and high stability against photo-corrosion when the surface is surrounded with N-rich InGaN^32,33^ enables the InGaN medium to split water a one-step photo-excitation, in other words, a single photocatalyst material. Additionally, with the geometrical properties of the InGaN nanosheets, rationally tailored p-type dopant (Mg) results in a large built-in electric field between two parallel surfaces separating the charge-carriers. As a result, the InGaN nanosheets minimize charge carrier recombination successfully which leads to a STH efficiency around 3.3%. It is noteworthy that InGaN nanosheets are either a photocathode or photoanode or a tandem structure of both photoelectrodes (*Z*-scheme) in stringent meaning. Yet, InGaN nanosheets achieve charge separation not by the intentional p–n scheme but by the rationally tailored p-type dopant; thus, the structure in the literature is called a photochemical diode (PCD).

**Fig. 3:**
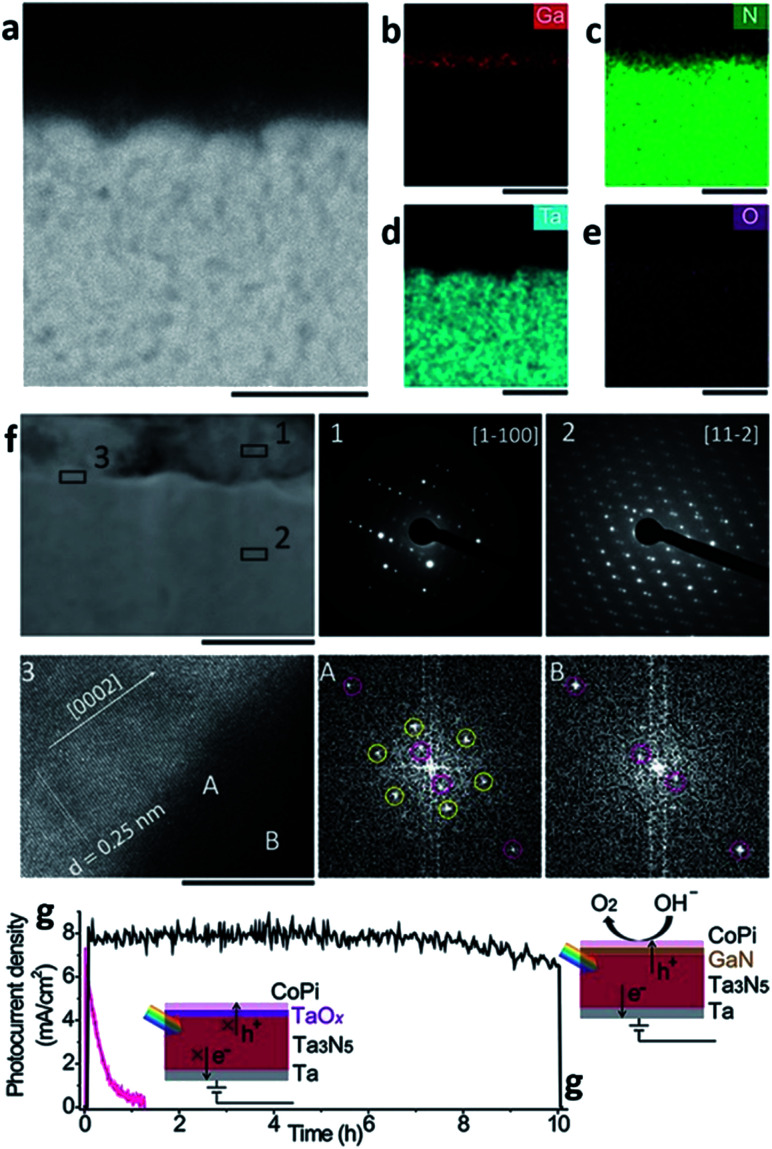
Reconstructed image from ref. 35. (a–e) Cross-sectional electron dispersive spectroscopy (EDS) mapping images of a GaN/Ta_3_N_5_ scanning transmission electron microscopy (STEM) image. The scale bar is 100 nm. (f) Cross-sectional STEM image of GaN/Ta_3_N_5_. The scale bar is 100 nm. Insets (1) and (2) are selected area diffraction (SAED) patterns acquired from the GaN region and Ta_3_N_5_, respectively. Inset (3) is a high-resolution transmission electron microscopy (HRTEM) image with the corresponding fast Fourier transform (FFT) patterns acquired at region A of the GaN/Ta_3_N_5_ interface and region B of the Ta_3_N_5_. The scale bar is 10 nm. (g) Time-course photocurrent density curves for the CoPi/GaN/Ta_3_N_5_ (black) and CoPi/Ta_3_N_5_ (pink) photoanodes. Reprinted with permission from ref. 35. Copyright 2017 John Wiley & Sons.

As mentioned above, MBE can ensure materials with high crystallinity, but it is an expensive process. The high cost comes not only from the system requirements but also from the substrate itself; the substrate to grow epitaxy with MBE should also be a single crystal which in turn results in a high fabrication cost for the PEC cell. PVD processes other than MBE typically do not need such requirements, and the sputtering process is one example. There are various routes for evaporating target materials to deposit a thin-film in the sputtering techniques such as direct current (DC), radio-frequency (RF), reactive, magnetron and their hybrids. However, magnetron sputtering is the dominant method nowadays.^34^ The sputtering uses atoms ejected as a result of momentum exchange when magnetically induced energetic ions collide with the target materials. It also requires a high-vacuum atmosphere, but the conditions are not as stringent as they are for the MBE system, and the substrate does not need to be a single crystalline thin-film. With the versatility of the target materials, the sputtering process is also a thin-film deposition technique widely used across a variety of fields including the deposition of photoelectrodes. Recently, Miao Zhong *et al.* reported on a photoelectrode with a Ta_3_N_5_ thin-film prepared with a sputtering and nitridation process (Fig. 3).^35^ Ta_3_N_5_ as a metal nitride offers wider absorption spectra than metal oxides and has more favorable band positions than other materials for solar applications such as group IV, III–V, and II–VI semiconductors. For these reasons, it has been studied intensively as a photoanodic material. However, poor stability in electrolytes was a main obstacle in utilizing Ta_3_N_5_ for PEC cells. As mentioned in the MBE section, nitrides containing gallium are stable against photo-corrosion^32^ but also resistive to electrolytes.^36^ Moreover, GaN forms a staggered band alignment when situated next to Ta_3_N_5_ and thereby suppresses charge recombination enhancing the overall performance of the PEC cell. CoPi/GaN/Ta_3_N_5_ photoanode comprised of sputtered Ta_3_N_5_ and electron beam evaporated GaN can achieve a high photocurrent density of 8 mA cm^−2^ and a hypothetical half-cell STH efficiency of around 1.5%.

**Fig. 4:**
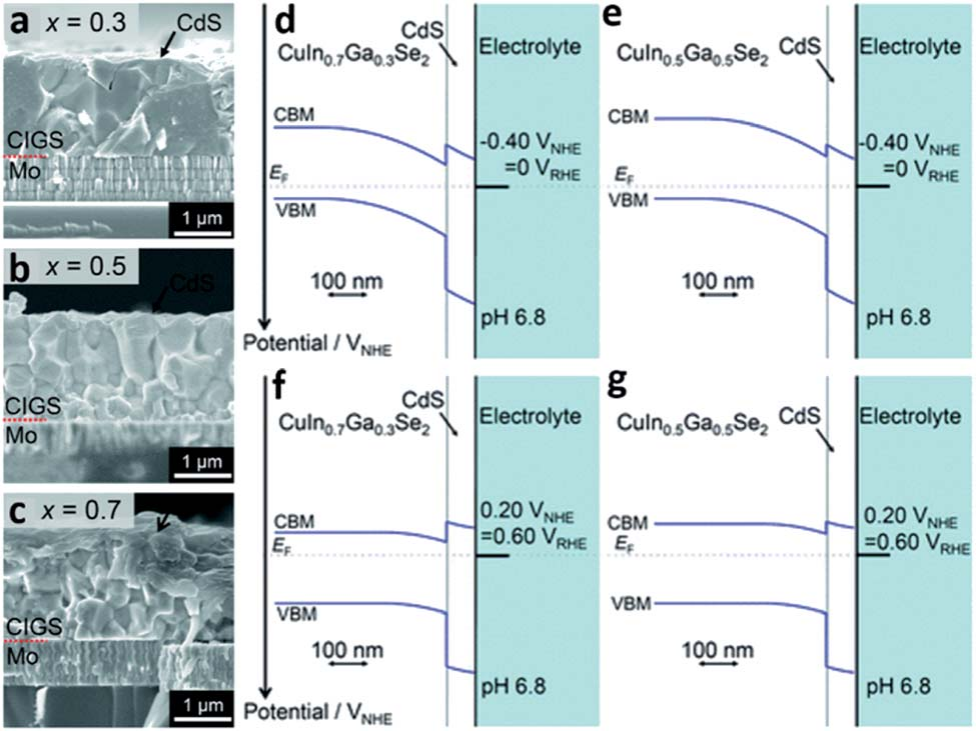
Reconstructed image from ref. 38. (a–c) Cross-sectional SEM images of the CdS/CIGS samples with a Ga/(Ga + In) ratio of 0.3, 0.5, and 0.7. Calculated band diagrams for the solid–liquid interfaces of the samples with a Ga/(Ga + In) ratio of 0.3 and 0.5 at an applied potential of *V*_RHE_ = 0 (d and e) and *V*_RHE_ = 0.6 (f and g) where RHE represents the reversible hydrogen electrode. Reprinted with permission from ref. 38. Copyright 2018 the Royal Society of Chemistry.

The evaporation process is another example of a method to synthesize PEC cells with the physical vapor deposition route. In this process, the target material is evaporated into a gaseous phase through thermal means under a high vacuum atmosphere. The distinguishable feature of the evaporation process compared with sputtering is evaporation based on thermal energy. In the sputtering process, atoms are ejected from the source or target materials at room temperature through the impact of gaseous ions.^34^ The main advantage of the evaporation process is the direct transfer of energy to the target material and thus, is very efficient in depositing thin-films. In turn, the evaporation process has a widely viable deposition rate from 1 nm min^−1^ to 1 μm min^−1^. There are various ways to heat the target materials such as electron beam, resistive heating, *etc.* Electron beam is the most common method of heating in the evaporation process and is typically used for metal contact deposition. However, when it comes to copper–indium–gallium–selenide (CIGS) photocathodic PEC cells, the thermal evaporation process with Knudsen cells is the mainstream technique. Thermal evaporation is also preferred over physical vapor deposition such as sputtering. This is because PEC cells made with the thermal evaporation process have better performance than the ones made with the sputtering process.^37^ Recently, H. Kobayashi *et al.* reported a STH efficiency of 3.7% from a modified CIGS photocathode and a BiVO_4_/Fe/Ni photoanode with the *Z*-scheme tandem configuration (Fig. 4).^38^ The CIGS photocathode was prepared with a three-stage method, that is, the layers of the CIGS photocathode were deposited using three different methods according to the materials used for the photocathode; the Mo back contact, CIGS light-absorber, and CdS buffer layers were formed by magnetron sputtering, high vacuum evaporation, and chemical bath deposition (CBD), respectively. CIGS is a promising candidate for PEC cells due to its long absorption edge wavelengths and its band structure which is suitable for solar-driven water splitting.^38,39^ With a decrease in the Ga/(Ga + In) ratio, the CIGS thin-film becomes columnar which can enhance the electrical conductivity. On the other hand, an increase in the Ga/(Ga + In) ratio results in a decreased CdS/CIGS interface which can enhance the diffusion of photoexcited electrons from the CIGS thin-film to the hydrogen sites. With the opposite behaviors of forming a columnar structure and enhancing the diffusion of photoexcited electrons as functions of the Ga/(Ga + In) ratio, a device structure with a GIGS thin-film with a carefully chosen Ga/(Ga + In) ratio of 0.4 exhibited the maximum cathodic current density.

### 2.2 Chemical vapor deposition

Another route to synthesize PEC cells is CVD. As briefly mentioned in Section 2.1 Physical vapor deposition, CVD is distinguished from PVD by the manner of chemical reaction in the synthesis or growth of the thin-films. While PVD utilizes material transfer from an evaporant of a condensed-phase or sputter target source, CVD relies on a vapor-phase converted from metals or mixture of chemicals by thermal energy under different gas conditions. In other words, the materials to be deposited on the substrate consequently come from the decomposition of volatile chemical precursors such as metal–organic compounds. One of the key advantages of the CVD process is the ability to deposit a thin-film using a large variety sources including metals, semiconductors, and even organic compounds. In addition, CVD techniques usually do not require a high vacuum atmosphere; thus, the system configuration is rather simple. This fact directly indicates that the deposition system and the operating expenses are in an affordable range, and this is another advantage to be gained by using the CVD process^34^ for the formation of PEC cells. With these features of the CVD process, variations of the CVD technique have been used based on the deposition conditions and the nature of the precursor including the following: atmospheric pressure CVD (APCVD), low-pressure CVD (LPCVD), plasma-enhanced CVD (PECVD), laser-enhanced CVD (LECVD), metal–organic CVD (MOCVD) and so on. Hydride vapor phase epitaxy (HVPE) and atomic layer deposition (ALD) are also categorized as CVD techniques as vapor-phase processes.

**Fig. 5:**
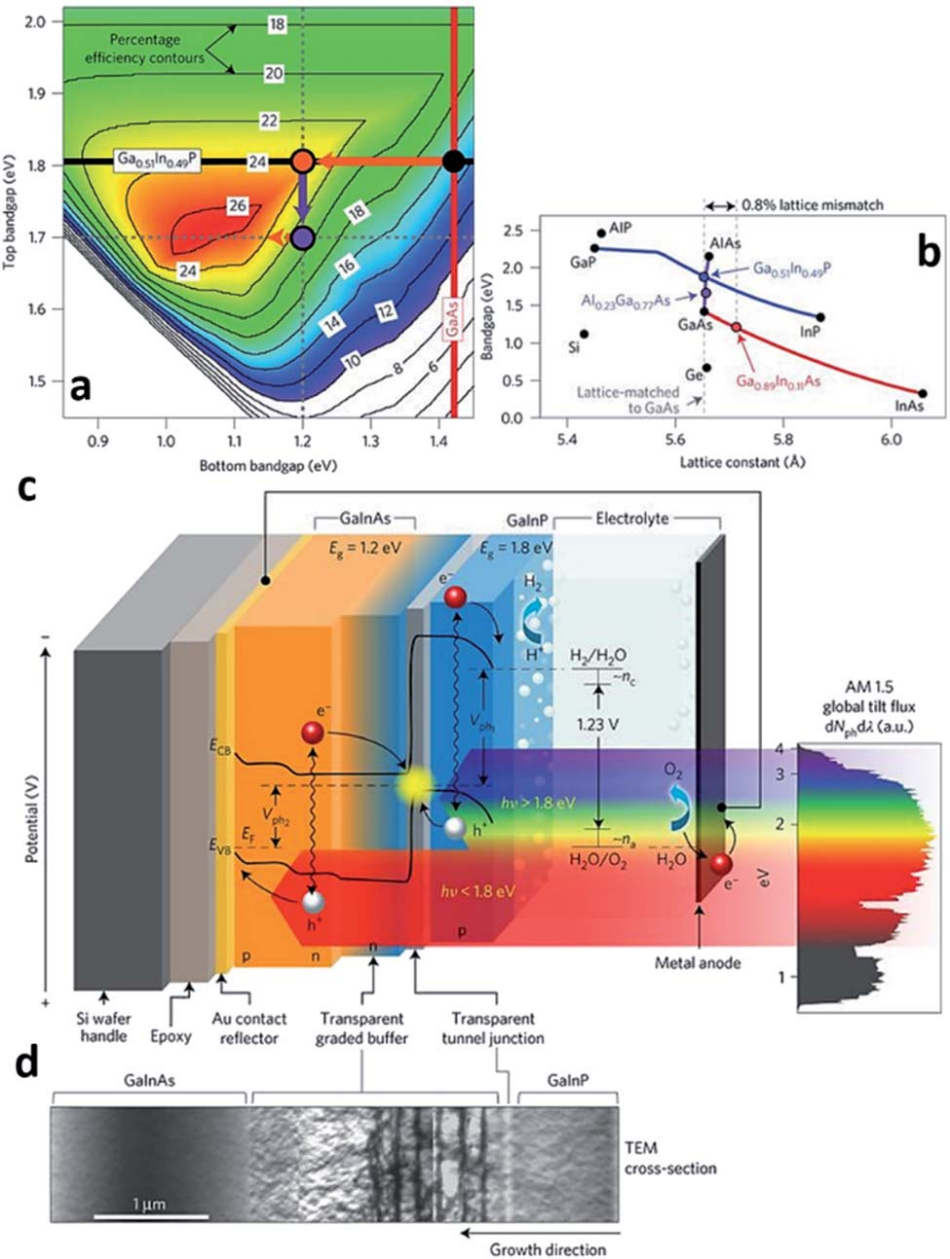
Reconstructed image from ref. 41. (a) Theoretically attainable efficiencies are plotted for the ranges of the top and bottom junction band gap energies. The values on the contour lines represent the STH efficiency attainable using the given top and bottom junction band gap energies. The 1.8/1.4 eV bandgap combination of the classical, lattice-matched GaInP/GaAs tandem could achieve a STH efficiency of 15% (black dot); 1.8/1.2 eV could achieve a STH efficiency of 24% (orange dot). (b) 1.8 eV GaInP and 1.2 eV GaInAs have a lattice mismatch around 0.8%. (c) and (d) represents schematics of the PEC cell and TEM image of CGB layer, respectively. Reprinted with permission from ref. 41. Copyright 2018 Springer Nature.

MOCVD also known as metal–organic vapor-phase epitaxy (MOVPE) is distinguished from the other CVD processes by the chemical nature of the precursors. The precursor gases such as trimethylgallium (TMGa) are metal–organic compounds and thus, easily decompose on the heated substrate in the absence of oxygen leaving atoms such as Ga from TMGa on the substrate surface. The atoms left on the sample surface then form a high crystalline thin-film with the other atoms (As) left by the hydrides such as arsine (AsH_3_) and/or another metalorganic compounds such as tertiary-butyl arsine (TBAs) in a kinetically limited growth regime.^40^ With the ability to grow high crystallinity materials, MOCVD is classified as an epitaxy growth technique along with MBE, HVPE, liquid-phase epitaxy (LPE), *etc.* Recently, James L. Young *et al.* reported a STH efficiency of 16.2% with a highly crystalline, inverted metamorphic GaInP/GaInAs tandem architecture grown by MOCVD (Fig. 5).^41^ The high efficiency of the PEC cell was possible because of the efficiently shared solar illumination flux by the stacked, series connected GaInP/GaInAs tandem absorbers by the virtue of their band gap energies. In other words, higher energy and lower energy photons are absorbed by the 1.8 eV top GaInP absorber and the 1.2 eV bottom GaInAs absorber, respectively. However, the lattice mismatch between the 1.8 eV GaInP and 1.2 eV GaInAs is 0.8% which could possibly limit the absorber thickness deteriorating the crystal quality and forming misfit dislocations inside the absorbers. As a way to overcome this issue, an AlGaInP compositionally graded buffer (CGB) layer was introduced between the 1.8 eV GaInP and the 1.2 eV GaInAs absorbers to confine the misfit dislocations inside the CGB layer. The CGB layer is not an illumination flux absorber, that is, an inactive component and transparent media to the photons having energy lower than 1.2 eV, thus the bottom GaInAs absorber can preserve light absorption. The photo-generated electrons then are transported to the top of the GaInP absorber, and the photocathode surface then splits the water producing hydrogen. One thing to note is, the sidewall and metal contact of the GaInP/GaInAs tandem absorber were surrounded by SU-8 photoresist to prevent the expected dark current when they come into direct contact with the electrolyte. Growth of the photocathodic PEC cell with MOCVD can ensure a high performance with a good material quality. Although MOCVD-grown PEC cells have a record-high STH efficiency, the fabrication cost is also very high. Expensive metalorganic sources, hydride sources and high purity hydrogen/nitrogen gases primarily account for the high fabrication cost. The requirement for a single crystalline substrate as in MBE also increases the cost. The high system complexity of MOCVD also increases the fabrication cost due to expensive maintenance.

**Fig. 6:**
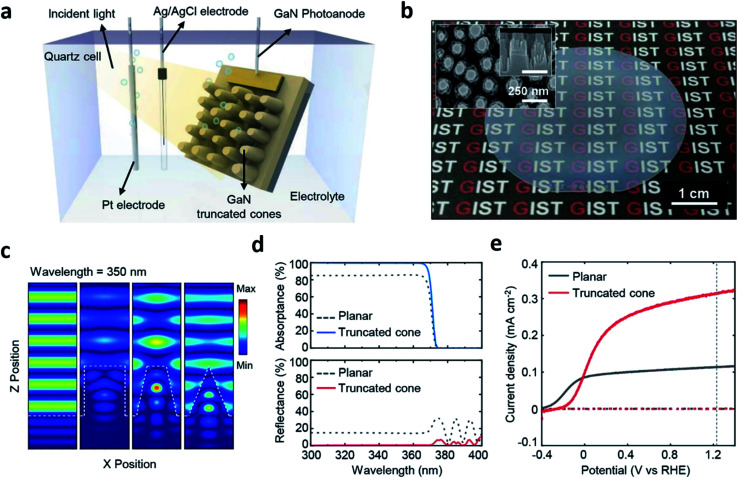
Reconstructed image from ref. 42. (a) A schematic diagram of the photoelectrochemical cell. (b) A Photograph and SEM image (inset) of GaN truncated nanocones on a 2-inch sapphire substrate fabricated by a 15 min. Etched SiO_2_ mask. (c) Three dimensional finite-difference time-domain simulations for the electric field distribution of the planar, cylindrical, truncated cone, and cone. (d) Simulation results of the absorptance/reflectance spectra of planar and truncated cone. (e) Photoelectrochemical measurements of the planar and truncated nanocones. Reprinted with permission from ref. 42. Copyright 2018, American Chemical Society.

Because the cell fabrication cost becomes inevitably high when it comes to PEC cells formed through MOCVD, other routes should be considered to reduce the cost of hydrogen fuel production. This can be resolved by enhancing the STH efficiency and using nano-structures in PEC cells that are suitable for water splitting which could be an alternative route.^42^ As briefly mentioned in the PVD section, structuring the PEC cell can enhance light absorption and surface reactions thereby can possibly increase the STH efficiency.^30^ However, the method addressed in ref. 30 is a bottom-up approach and is not an easy way to precisely tailor a structure that is optimized for PEC cells despite the surface area can be increased for chemical reactions. Furthermore, quite different to the light management in other applications such as light emitting diodes, photovoltaic cells, functional glasses, *etc.*,^43–45^ designing nano-structures for PEC cells is rather complicated due to the photoelectrochemical nature. There have been some efforts on structuring PEC cells using this approach.^46,47^ However, most of the studies simply focused on optimizing the structure from the perspective of surface reflection, which is not different from the other non-PEC cell devices. Yeong Jae Kim *et al.* reported on a top-down structured MOCVD-grown n-type GaN photoanode and computational methods with a finite difference time domain (FDTD) and a rigorous coupled wave analysis (RCWA) for four different shapes, planar, rod, truncated cone and cone (Fig. 6).^42^ The study theoretically and experimentally revealed that the truncated cone is the most appropriate for light trapping on the nanostructure close to the electrolyte reducing the path of photo-generated charges, in other words, a fast charge transport, and consequently showed an increase in the photocurrent. Furthermore, thermally dewetted metal particles for top-down structuring of a PEC cell with a truncated cone shape is a controllable and non-lithographical approach and thus, can reduce the fabrication cost. A MOCVD-grown high quality photoanodic material, metal particle-assisted easy top-down structuring, and a precisely designed truncated cone shape suited for PEC cells can possibly reduce the hydrogen fuel production cost and the cell fabrication cost.

**Fig. 7:**
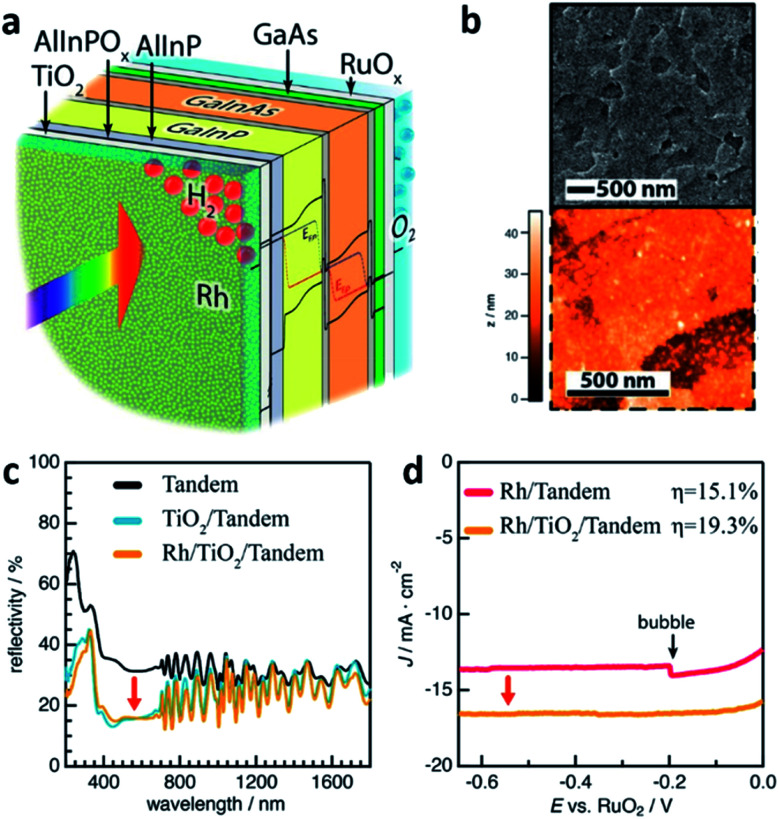
Reconstructed image from ref. 49. (a) Schematic of the GaInP/GaInAs tandem photoelectrode after functionalization with an interfacial TiO_2_ thin-film and Rh electrocatalysts. (b) Topography of the Rh-particle-coated crystalline anatase TiO_2_ layer by SEM and AFM. (c) Reflectivity of the GaInP/GaInAs tandem photoelectrode without ARC (black curve); second reflectivity obtained after anatase TiO_2_ thin-film deposition (blue) and after photoelectrochemically deposited Rh nanoparticles (yellow). All reflectivity obtained in the air. (d) Output characteristics of the PEC cells displaying effect of anatase-phase TiO_2_. Reprinted with permission from ref. 49. Copyright 2018, American Chemical Society.

ALD also called atomic layer epitaxy (ALE) differs from other CVD techniques by its thin-film formation mechanism which is a surface catalytic reaction or self-limiting surface chemical reaction. Precursor reacts with the substrate as a result of a surface catalytic reaction and leaves no more than one monolayer (ML) because the surface reaction is completed by covering the substrate surface. This feature enables the ALD technique to be used as a tool for a precise layer-by-layer deposition process.^48^ In the manner of a self-limiting growth regime and layer-by-layer growth, ALD seems similar with MOCVD. However, instead of flowing all precursor gases at the same time in MOCVD and in a continuous flow, the precursor gases are supplied one-by-one without overlapping pulses in an alternating fashion on the substrate surface.^34^ Depending on reaction mechanisms of each individual ALD method, a variety of processes are available such as the deposition of metals, nitrides, sulfides, chalcogenides, and so on. The key advantage of ALD is the precise control of the thin-film thickness with the help of the self-limiting surface reaction as mentioned above. A relatively low growth temperature due to the use of a catalytic reaction on the substrate surface is also another advantage of using the ALD process. However, the advantage, in turn, becomes a disadvantage. Due to the layer-by-layer deposition on an atomic scale, the deposition rate is very low which is 1 mL per cycle in principle. Yet, ALD could form thin-film not only on planar but also on nanostructured surfaces with a high atomic density thus a good tool to protect the layer beneath. In this manner, ALD thin-films are frequently used for a top-coat layer for PEC cells. W.-H. Cheng *et al.* reported the world highest STH efficiency of 19.3% (Fig. 7).^49^ The structure of their PEC cell consisted of MOCVD-grown multiple stacks of absorbers sharing the incident solar flux and is similar to the one reported by James L. Young *et al.*^41^ However, the PEC cell used a 30 nm-thick crystalline anatase-phase TiO_2_ photocathode interfacial layer which was deposited by ALD to facilitate a reduced reflectivity and interface recombination velocity. TiO_2_ is a good protective layer against corrosion naturally and reduces the reflectivity by 15% with the anatase-phase. In addition, the TiO_2_ interlayer has a good energy band alignment with the window layer of the tandem absorber and an interfacial ultra-thin oxidized surface with the electrolyte. In detail, photo-generated electrons, which are minority carriers in the main part of the absorbers, become majority carriers when they travel to the AlInP and TiO_2_ interlayer. The carriers then experience reduced recombination losses during the carrier transport with the help of the conduction band alignment between AlInP and TiO_2_. In addition to what has already been discussed, ALD also can be used to dope a host material. In a recent report by Guru Dayal *et al.*, ALD was used to deposit a Sn layer.^50^ The Sn layer then diffused into and doped the solution-processed hydrothermally grown FeOOH nanorods. The main reason for using ALD to dope FeOOH nanorods with Sn is for conformal doping around the nanorods, that is, uniform diffusion of Sn throughout the nanorods, consequently leading to the successful transformation of the Sn-surrounded FeOOH nanorods into uniform Sn-doped α-Fe_2_O_3_ (hematite) nanorods by an annealing process. As a result, a coupled form of a CH_3_NH_3_PbI_3_ (perovskite) light absorber and hematite nanorod photoanode had a STH efficiency of 3.4%.

HVPE is another CVD technique similar to MOCVD. However, in contrast to MOCVD, HVPE is governed by a near-equilibrium process which is controlled by the supersaturation of reactants on the substrate surface, not by a kinetically limited growth regime during the deposition of the thin-film.^40^ In typical HVPE, HCl reacts with group-III metals at elevated temperatures under atmospheric pressure and produces volatile metal chlorides. The gas then reacts with group-V hydrides such as NH_3_ and forms a V–III-Cl complex on the substrate. Next, the metal complex decomposes on the heated substrate surface forming a thin-film finally. HCl, which is regenerated after the chemical reaction on the surface, consequently, has to be vented out. With the near-equilibrium process, the deposition rate of HVPE grown thin-films can be very fast in the range of 1–100 μm h^−1^ depending on the precursor supersaturation or mass transport to the substrate surface. Along with the fast deposition, a huge reduction in the fabrication cost with the help of cheaper source materials is the biggest benefit when using HVPE. However, some difficulties such as abrupt junction formation and metal chloride control prevent HVPE to be a mainstream technique for thin-film formation. Recently, with the importance of the device fabrication cost, HVPE has been revisited for low-cost and high-efficiency III–V compound semiconductor tandem solar cells.^51^ A. Martinez-Garcia *et al.* reported on a HVPE-grown GaSb_*x*_P_1−*x*_ photoanodic PEC cell.^52^ Without introducing a complex structure such as nanoparticles, nanowires, and/or multiple-stacked absorbers, the GaSb_*x*_P_1−*x*_ PEC cell was fabricated in a simpler form as a bulk GaSb_*x*_P_1−*x*_ crystal covered with ML-thin IrO_2_. In the study, a Si (100) substrate with a 3° miscut was used to form a 150–200 μm-thick GaSb_*x*_P_1−*x*_ film. The thin-film then was delaminated to ultimately obtain the free-standing GaSb_*x*_P_1−*x*_ bulk crystal. Incorporating Sb in GaP can tailor the band gap energy dramatically from 2.7 eV GaP to 0.7 eV GaSb and thus, can provide a proper strategy for designing a band gap energy and band alignment that are well suited for solar-driven water splitting. Furthermore, the absorption coefficient of GaSb_*x*_P_1−*x*_ by adding Sb is 30 times higher than that of the pure GaP for photon energies higher than the direct transition energy, 2.7 eV. Above the indirect transition energy, the absorption coefficient is three times larger for the GaSb_*x*_P_1−*x*_ than for the pure GaP with a presumable interaction between the p-orbitals of Sb and the valence band of GaP resulting in an increase in the density of states at energies greater than 2.68 eV.^53^ The enhanced absorption coefficient and other additional effects occurred by adding 3 at% of Sb to GaP, which eventually resulted in a STH efficiency of 2%, and is expected to be increased further by enhancing the crystal quality.

Other than the above-mentioned techniques, many CVD methods have been used to fabricate the PEC cells currently available. However, even with the rather low fabrication cost because of the relaxed requirements such as a low vacuum level and a simpler system configuration than that of PVD systems, it still has a high cost yet for the fabrication of PEC cells. From this perspective, methods that do not require a vacuum system and/or an expensive gas transportation system such as electrochemical deposition (ECD), photo-electrochemical deposition (PECD), and CBD should be beneficial.

### 2.3 Electrochemical deposition

**Fig. 8:**
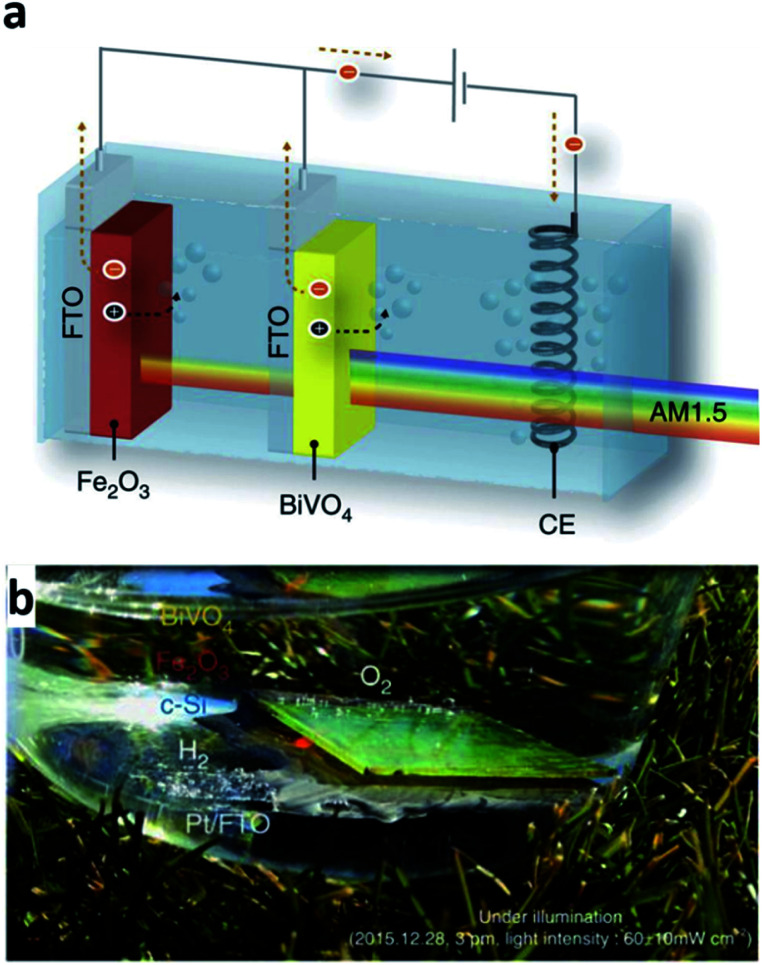
Reconstructed image from ref. 58. (a) Scheme of a tandem cell with a hetero-type dual photoelectrode (BiVO_4_/Fe_2_O_3_) and parallel-connected Si solar cells (crystalline Si in parallel connection), (b) artificial leaf (monolithic tandem cell) in action under illumination with real sunlight. Reprinted with permission from ref. 58. Copyright 2016 Springer Nature.

One clear difference with electrochemical deposition (ECD) compared with the other techniques mentioned above is the absence of a vacuum during the deposition process. This is because in the ECD process, the ions dissolved in the liquid electrolyte are the source of the thin-film deposition. In other words, the ions in the electrolyte are reduced and deposited on the electrode surface when the appropriate voltage is applied to the system; thus, the entire thin-film deposition proceeds in the electrochemical cell. The electrochemical cell consists of four elements: a working electrode, counter electrode, reference electrode, and electrolyte. The working electrode is where the reaction of interest occurs, that is, the positive ions in the electrolyte are deposited on the surface of the working electrode. The reference electrode enables the accurate control of the potential of the working electrode, and the counter electrode is the element completing the electric circuit and enables the current flow. A two-electrode cell without the reference electrode is used for ECD frequently. In the two-electrode system, however, the potential of the working electrode is measured against the counter electrode. Hence, the measured voltage depends on the current density and electrolyte concentration, which thereby can possibly result in a poor quality and inhomogeneous deposition.^54^ As mentioned, the key advantage of the ECD process is its low-cost route to form thin-films due to a simple system configuration. The simplicity of the deposition set-up also has another benefit such as a possible broad implementation of the method with a variety of variations.^14^ For example, photo-electrochemical deposition (PECD), an ECP process, is a method using incident photon flux to acquire a uniform facet coverage and is one of the stable ECD processes. Another advantage of ECD is scalability. With the low STH efficiency of the current PEC cells technologies still, the ability to form a large area PEC cell could be an important issue. From this perspective, using a large capacity tank can simply provide a solution when it comes to the ECD process.^55^ However, this method is available only if the target substrate to deposit is conductive due to its own deposition mechanism. Additionally, ECD processed thin-films are typically polycrystalline or amorphous.^56^ In addition, the processed thin-films have a role not as a core medium absorbing incident light for water splitting but as an additional component to extend the functionality of other core materials such as BiVO_4_ and Fe_2_O_3_ which are formed by methods other than by the ECD process.^57,58^ For example, the optimized ECD deposition of Fe(Ni)OOH on the nanoscale cone-shaped-Si/BiVO_4_:Mo photoanode resulted in the enhancement of the photocurrent for water oxidation.^57^ BiVO_4_ reported in that article is indeed known as a promising photoelectrode material due to its band gap energy around 2.4 eV and having an adequate conduction band edge position with respect to the H_2_O/H_2_ evolution level.^59^ The ECD processed Fe(Ni)OOH coating on the photoanode further enhanced the functionality with a cathodic shift and rapid photocurrent. The cone-shaped-Si/BiVO_4_:Mo/Fe(Ni)OOH photoanode exhibited a STH efficiency of 6.2% consequently. As another example, PECD processed NiOOH/FeOOH co-catalysts on the BiVO_4_ surface of a BiVO_4_-hematite hetero-type dual photoelectrode enhanced the charge carrier injection efficiency to the electrolyte with an improved oxygen evolution in combination with Ni_2_FeO_*x*_ and TiO_2_ on hematite (Fig. 8).^58^ The hetero-type dual photoelectrode, as a result, achieved a STH efficiency of 7.7%. In another example, an ECD processed metal film also has a role as a catalyst in the PEC cell. Recently, Ibadillah A. Digdaya *et al.* reported on an a-SiC photocathode deposited with a Ni–Mo dual catalyst.^60^ With the incorporation of carbon, the band gap energy of a-Si can be tailored in the range of 1.8–2.1 eV.^61^ The tunability of the band gap energy of a-SiC, in turn, can be used in a tandem PEC cell for maximum light availability. In addition, the absorption coefficient of a-Si is higher than that for c-Si by three orders in the UV and visible spectral range with a lower cost.^62^ However, poor stability when it comes to aqueous environments was the main issue with the availability of a-Si as a photoelectrode. To protect the a-Si surface, anti-corrosive TiO_2_ was typically used which effectively passivates the a-Si photoelectrode. However, the TiO_2_ surface is not that much active in the hydrogen evolution reaction in this case. As an alternative, a dual-catalyst formed with a Ni thin-film and Ni–Mo nanoparticles was introduced on the TiO_2_/a-Si photoelectrode in another study. Basically, Ni and Ni–Mo are also persistently strong in an alkali electrolyte^63^ providing room to increase the pH of electrolyte further, which thus can reduce ohmic loss. Furthermore, an ECD processed Ni thin-film on the top-most surface can promote more active sites due to the fact that Ni is an active electrode for the hydrogen evolution reaction in an alkaline environment. Through a combination of the effects and enhancement of the kinetics, the Ni–Mo/Ni/TiO_2_/a-Si photoelectrode successfully enhanced the photocurrent density.

### 2.4 Other methods

Other than the methods mentioned above such as the PVD, CVD and ECD processes, there are other routes to form materials for water splitting thereby defined as PEC cells. The hydrothermal process is one of the methods to synthesize a crystalline material in aqueous solution under high temperature and high pressure depending on the solubility of the substances.^64^ The hydrothermal process does not require a complicated configuration and thus, can be used across various applications.^65^ Key advantages of the hydrothermal process are a fast chemical reaction and the ability to form highly dispersive substances in solution during synthesis which can result in a uniform crystallinity throughout entire area. However, an autoclave, a steel pressure vessel, is necessary for the synthesis but expensive yet though far cheaper than other well-known crystal growth tools such as MBE, MOCVD and HVPE. Peng Yi Tang *et al.* reported on a hydrothermally grown hematite nanowire photoanodic PEC cell.^66^ Hematite, as briefly mentioned in the CVD section, is a promising photoanodic material with its own properties suitable for water splitting including photo-chemical stability, a tunable band gap energy in the range of 1.9–2.2 eV, and a high theoretical STH efficiency around 15.4%.^67,68^ Nevertheless, the low absorption coefficient, short carrier lifetime, low oxygen evolution reaction kinetics, short hole diffusion length, poor electrical conductivity and their resulting multiple carrier recombination pathways throughout whole structure make hematite difficult to achieve the expected theoretical STH efficiency.^68^ To avoid such performance degrading pathways, it is necessary to secure a rapid charge transport and transfer from the photoanode to the back substrate and *vice versa*. In the report by Peng Yi Tang *et al.*, indium-tin-oxide (ITO)/hematite/Fe_2_TiO_5_/FeNiOOH multi-layered nanowires grown on a fluorine-doped tin oxide (FTO) substrate were suggested as a solution to achieve this purpose, and this hydrothermal process was adapted to grow hematite nanowires on ITO coated FTO substrate.

Spray deposition, also known as spray forming or spray casting is another route to fabricate PEC cells. Spraying semi-solid droplets onto a heated substrate defines the material deposition. The semi-solid droplets typically are prepared by an induction furnace and sprayed through a spray gun to a ceramic nozzle with a compressed carrier gas.^69^ This method is a simple and low-cost solution for deposition onto a large area surface. With these advantages, it is suitable for mass-production. Moreover, it is possible to control the composition and tailor the microstructure formed on the substrate surface with the spray deposition. However, the surface can be easily contaminated depending on system configuration. Furthermore, long-time spraying can possibly clutter the nozzle.^70–72^ Yongsheng Guo *et al.* reported on a ZnFe_2_O_4_ (zinc ferrite) photoanode formed through spray deposition.^73^ Quite similar to other photoanodic materials, zinc ferrite is also regarded as a candidate material for a highly efficient water splitting PEC cell with its own narrow band gap energy of around 1.9 eV and expected high STH efficiency close to 20%.^74^ In the study, dissolved zinc acetate and ferric acetylacetonate at a molar ratio of 1 : 2 in methanol were used to prepare zinc ferrite precursor. The precursor then was sprayed onto a heated FTO substrate at 400 °C for 45 min. Subsequently, the film deposition was defined with calcination of the spray deposited thin-film in air under a heated atmosphere, which resulted in an enhanced photocurrent density.

Table 1 shows noticeable parameters and configurations of the selected representative PEC cells mostly discussed in this article. It is note that detailed configurations of some PEC cells are not clear due to the lack of information provided in the literatures. Regarding the methods listed in the table, there are many other routes to fabricate PEC cells except for the methods mentioned above. For example, a film formed through Langmuir–Blodgett was also used to form water splitting electrodes.^80^ However, needless to say, just a single material synthesis or growth method alone cannot define the PEC cell. Even the simplest water splitting photoanode introduced in this article, for example, HVPE-grown GaSb_*x*_P_1−*x*_, requires an IrO_2_ thin-film which is prepared by a solution-based process for a properly working photoanode.^51^ Balancing the fabrication cost and STH efficiency of PEC cells has always been a key issue, and it largely rely on synthesis or growth methods as much as on precursor and/or source materials. From these perspectives, choosing a proper method based on the component layer and/or structure of the PEC cells keeping in mind the cost-performance balance would lead to the best strategy for achieving a high performance, low cost PEC water splitting cell.

**Table 1:** Photoelectrode configurations, types, synthesis/growth methods, electrolytes, and STH efficiencies of several representative PEC cells. Tunnel junction which connect two different photo-absorbers or solar cells, and details of individual photo-absorbers and/or solar cells are not addressed in the table below. For the detailed structure, see the references. Reprinted with permission from ref. 41. Copyright 2016 Springer Nature, ref. 49. Copyright 2018 American Chemical Society, ref. 75. Copyright 2015 John Wiley & Sons, ref. 75. Copyright 2016 Elsevier Inc, ref. 35. Copyright 2017 John Wiley & Sons, ref. 52. Copyright 2018 John Wiley & Sons, ref. 57. Copyright 2016 American Association for the Advancement of Science, ref. 77. Copyright 2017 Springer Nature, ref. 78. Copyright 2016 Springer Nature ref. 58. Copyright 2016 Springer Nature, ref. 79. Copyright 2017 Springer Nature, ref. 38. Copyright 2018 Royal Society of Chemistry, ref. 30. Copyright 2018 Springer Nature

Type	Configuration (layer(s)‖substrate‖layer(s))	Method	Electrolyte (pH)	*η*_STH_ (%)	Reference
Photocathode	Si‖epoxy/Au/GaInAs/AlGaInP CGB/GaInP/PtRu	MOCVD	3 M H_2_SO_4_ (1.0)	16.2	41
Photocathode	RuOx‖GaAs‖GaInAs/GaInAs CGB/GaInP/AlInP/TiO_2_/Rh	MOCVD	1 M HClO_4_ (0)	19.3	49
Photocathode	FTO‖Au/Cu_2_O/AZO/TiO_2_RuO_2_/TiO_2_	ECD	0.5 M Na_2_SO_4_ + 0.1 M phosphate (5.0)	2.5	75
Photocathode	Glass‖ZnO:Al/a-Si:H/a-Si:H/μc-Si:H/μc-Si:H/ZnO:Al/Ag/Pt	PECVD	0.1 M KOH (13)	7.8	76
Photoanode	Ta‖Ta_3_N_5_/GaN/CoPi	Sputtering	0.5 M MKPi (13)	1.5	35
Photoanode	Si (delaminated)‖GaSbP/IrO_2_	HVPE	1 M H_2_SO_4_ (0.3)	2.0	52
Photoanode	Nanocone-FTO glass‖SiO_*x*_/Pt/SnO_2_/BiVO_4_:Mo/Fe(Ni)OOH connected with perovskite solar cell	ALD	0.5 M KH_2_PO_4_ (7.0)	6.2	57
Photoanode	FTO glass‖BiVO_4_/Ag/reduced-graphene-oxide	Hydrothermal	0.5 M Na_2_SO_3_ (6.8)	0.9	77
Tandem configuration	Pt/2-pair dense/porous ITO DBR/3-pair TiO_2_–SiO_2_ DBR‖FTO glass‖WO_3_‖BiVO_4_:Mo + dye/TiO_2_‖FTO glass‖Pt	Sputtering and solution processes	N/A (6.9)	7.1	78
Tandem configuration	FTO glass‖BiVO_4_:Mo/Fe(Ni)OOH + FTO glass‖α-Fe_2_O_3_/TiO_2_/Ni_2_FeO_*x*_ + Al‖c-Si solar cell‖Ag	ECD	1.0 M KCl (9.2)	7.7	58
Tandem configuration	IrO_*x*_·*n*H_2_O/Au/Pt/Ti/Pt/GaAs solar cell/AuGe/Ni/Au‖glass + Pt/Au/Ni/AuGe/GaAs solar cell/Si_3_N_4_–Pt/Ti/Pt/Au‖glass	MOCVD	0.5 M H_2_SO_4_ (0.55)	13.1	79
Tandem configuration	Mo-coated soda-lime glass‖CIGS/CdS/Pt + ITO-glass‖BiVO_4_/Fe/Ni	Evaporation	0.5 M K_3_BO_3_ + KOH (9.5)	3.7	38
Photochemical diode	Si‖InGaN:Mg/Rh/Cr_2_O_3_	MBE	N/A (7.0)	3.3	30

## 3. Summary

With the aim to develop a renewable and sustainable energy source in place of fossil fuels, solar-driven PEC cells as a method to produce hydrogen fuel with near-zero carbon emission has provided the way for several decades to improve the STH efficiency rapidly up to 19.3% per cell and over 30% on a system scale.^81^

However, balancing the fabrication cost and performance still is not sufficient. The United States Department of Energy established the target performance metrics of PEC cells^82^ as follows: hydrogen fuel cost below 2 dollars per kg, STH efficiency >25%, and PEC electrode cost less than 100 dollars per m^2^ with a 10 years lifetime. However, despite the fact that PEC cells can only meet economical requirements if the STH efficiency is higher than 10%, most of the PEC cells exhibit a poor efficiency. It is true that there are few PEC cells that have a STH efficiency much higher than 10%, and the ones that do are mostly formed thorough expensive methods such as MBE or MOCVD with a single crystalline multiple layer or absorber structure on a single crystalline substrate. According to the current status, PEC cell technology still has a long way to go, and forming suitable materials for PEC hydrogen production is presumably the foremost target to achieve. From this perspective, we have reviewed PEC cell technologies in terms of the growth and synthesis methods to provide guidance on research for solar-driven water splitting PEC cells.

## Conflicts of interest

There are no conflicts to declare.

## Supplementary Material
